# Breast Cancer Metabolomics: From Analytical Platforms to Multivariate Data Analysis. A Review

**DOI:** 10.3390/metabo9050102

**Published:** 2019-05-22

**Authors:** Catarina Silva, Rosa Perestrelo, Pedro Silva, Helena Tomás, José S. Câmara

**Affiliations:** 1 CQM - Centro de Química da Madeira, Universidade da Madeira, Campus Universitário da Penteada, 9020-105 Funchal, Portugal; cgsluis@staff.uma.pt (C.S.); rmp@staff.uma.pt (R.P.); pedro_dasilva@hotmail.com (P.S.); lenat@staff.uma.pt (H.T.); 2 Faculdade de Ciências Exactas e Engenharia, Universidade da Madeira, Campus Universitário da Penteada, 9020-105 Funchal, Portugal

**Keywords:** breast cancer, omics, analytical platforms, chemometric methods

## Abstract

Cancer is a major health issue worldwide for many years and has been increasing significantly. Among the different types of cancer, breast cancer (BC) remains the leading cause of cancer-related deaths in women being a disease caused by a combination of genetic and environmental factors. Nowadays, the available diagnostic tools have aided in the early detection of BC leading to the improvement of survival rates. However, better detection tools for diagnosis and disease monitoring are still required. In this sense, metabolomic NMR, LC-MS and GC-MS-based approaches have gained attention in this field constituting powerful tools for the identification of potential biomarkers in a variety of clinical fields. In this review we will present the current analytical platforms and their applications to identify metabolites with potential for BC biomarkers based on the main advantages and advances in metabolomics research. Additionally, chemometric methods used in metabolomics will be highlighted.

## 1. Introduction

**Figure 1:**
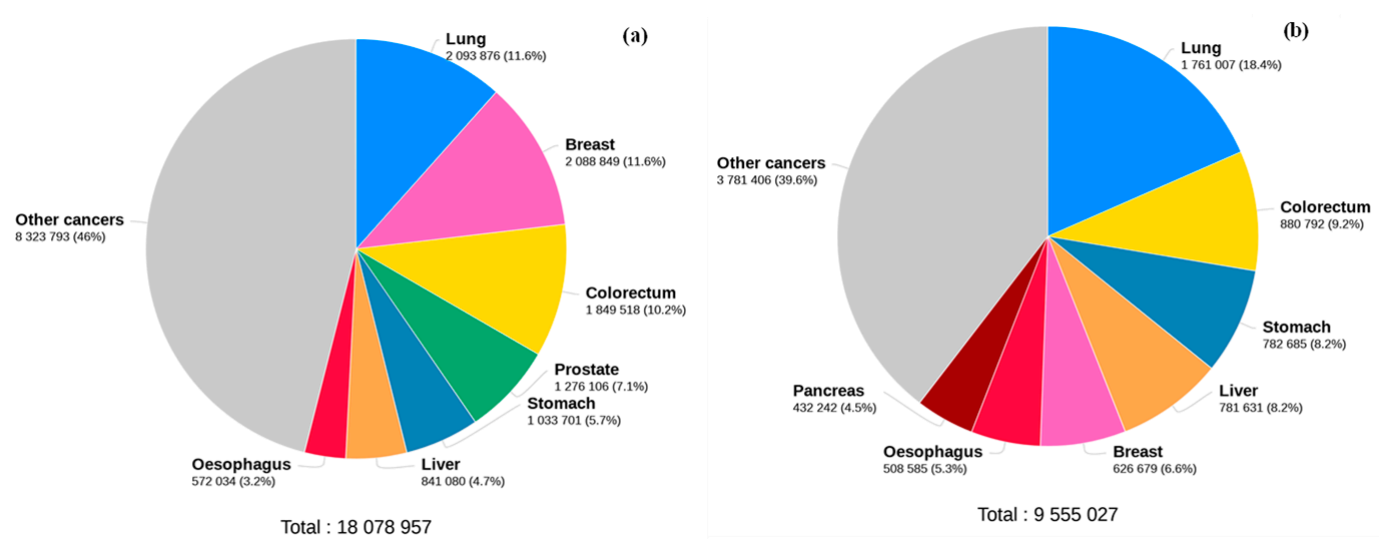
Estimated cancer incidence rates (**a**) and (**b**) estimated number of deaths worldwide for 2018. Adapted from GLOBOCAN [2].

Cancer is a public health problem and causes a tremendous burden on patients, families and society creating a significant problem on global economy. Although has been extensively investigated, cancer still remains one of the leading causes of death in the world after coronary diseases [1]. Globally, breast cancer (BC) remains at the top of women’s cancers worldwide followed by colorectal, lung, cervix, and stomach cancers according to GLOBOCAN series of the International Agency for Research on Cancer (IARC), contributing with more than 11.6% of all cancer types (Figure 1).

In addition, around 2.1 million BC new cases were diagnosed in 2018 and occurred 630 thousand deaths (6.6% of all cancers) (Figure 1b).

**Figure 2:**
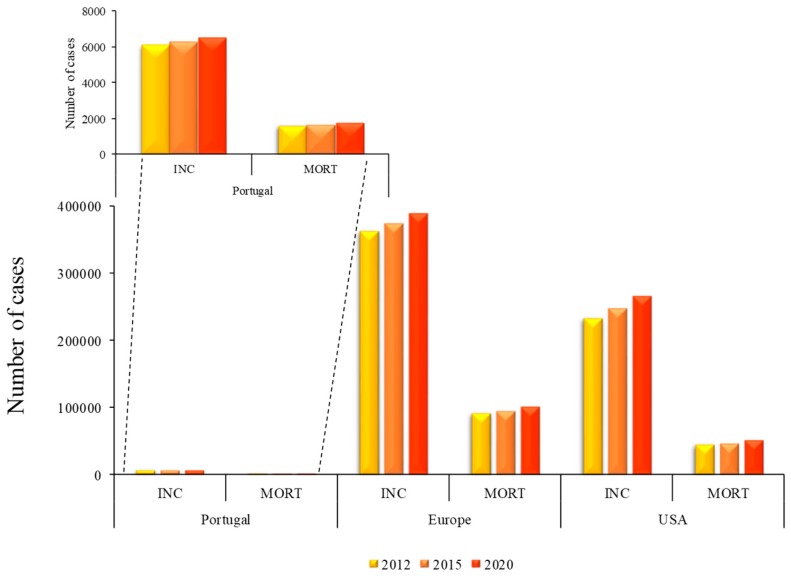
BC incidence and mortality rates in Portugal, Europe and USA from 2012, 2015 and expected rates for 2020. Data available at IARC. *Legend*: INC: incidence; MORT: mortality.

The incidence rates are highest in North America, Australia and Europe and lowest in Asia. These differences might be related to societal changes, as result of industrialization, such as, unhealthy lifestyle, expressed by overweight and other symptoms, alcohol consumption, tobacco smoking, physical inactivity, early menarche, among others [2,3]. Although the incidence is high in some developed countries, mortality is higher in low and middle income countries [4]. The incidence of breast cancer increases with age and is usually diagnosed in the 50–60 age group. Moreover, the most aggressive type of the disease predominates in the younger age group (below 35 years) whereas in the older age group (above 75 years), the treatment cannot be so aggressive and has to be adjusted [5]. Concerning the incidence rates and mortality for breast cancer in Europe, it was observed that in 2012, the incidence of breast cancer was around 361,608 cases with 91,585 deaths. For 2020, around 400 thousand new cases will be diagnosed resulting in 100 thousand deaths according to International Agency for Research on Cancer (IARC). For Portugal and USA the expected number of breast cancer cases in 2020 will be nearly 6000 and 270 thousand resulting in around 1700 and 51,000 deaths, respectively as shown in Figure 2.

This trend might be as consequence by the availability of better screening procedures resulting in an early detection and also in the development of new treatments [3,6,7], which lead to an improved survival. Several risk factors associated with BC have been already recognized, namely epidemiological factors (e.g., age, reproductive factors, socioeconomic status, ethnicity), often using standard analysis approaches (e.g., logistic regression) with adjustment for multiple comparisons. Other factors as lifestyle (e.g., alcohol, tobacco, obesity, physical activity), and exposure to radiation [8] are also associated. The risk of developing BC increases with age being rare in women younger than 25 years, but tending to be more aggressive in younger people. The most common BC that occurs is the invasive type independently of age [9]. The highest risk of family history is associated with increasing number of first-degree relatives diagnosed with BC (age under 50 years). The risk is further increased when the affected relative is diagnosed in both breasts [10]. Particularly, the mutations in genes BRCA1, BRCA2 and TP53 are strongly associated with the development of BC [9], even if these mutations are low, accounting for a small portion of the total BC incidence [2]. Consistent physical activity has many benefits and greater activity has been related to lower BC risk by decreasing the circulating estrogen levels in postmenopausal women [11,12]. Extensive literature has linked alcohol consumption to BC risk and reveal the role of ethanol in carcinogenesis altering estrogen levels through acetaldehyde. Briefly ethanol is converted to acetaldehyde (AA) through alcohol dehydrogenase (ADH), that then binds to DNA interfering with the DNA synthesis and repair [13]. Obesity is another BC risk factor to take into account as it is involved in insulin resistance and hyperinsulinemia [14]. Insulin has anabolic effects on cellular metabolism and an overexpression of insulin receptor has been demonstrated in human cancer cells [9,15]. The involvement of insulin-like growth factor (IGFs) in carcinogenesis is attributed to their role in linking high energy intake, increased cell proliferation, and suppression of apoptosis to cancer risks [15,16]. With regard to obesity and BC risk, some studies indicate that is strongly associated with increased invasive BC risk in postmenopausal women particularly for estrogen receptor–positive cancers (ER^+^) [17,18,19]. In clinical practice, there are nowadays several biomarkers routinely used for prognosis and identification of tumors, including the estrogen receptor (ER), progesterone receptor (PR) and the human epidermal growth factor receptor-2 (HER2) [20,21]. Another promising prognostic and predictive biomarker of BC is Ki-67 (present in dividing cells) as indicator of cell proliferation and also as an endpoint for neoadjuvant systemic therapy [20]. However there are other proposed markers of proliferation measured by immunohistochemistry (IHC), such as, cyclin D, cyclin E, p27, p21, among others that are used to determine the predictive and prognostic levels [22].

In the last years, metabolomics emerged as a powerful approach in the advanced disease biomarker discovery which includes the comprehensive study of metabolites that are present in biological samples [23]. The study of metabolome to search biomarkers for any disease involves the identification of endogenous metabolites that have the potential to discriminate between samples obtained from healthy subjects and diseased patients. Plasma, serum, urine, tissue and cerebrospinal fluid (CSF), are the most commonly used biological samples in metabolomic studies. These biological samples contain hundreds of metabolites that vary in chemical and physical properties and concentration levels. Metabolomic studies includes two main approaches – targeted and untargeted. The targeted analysis is focused in specific groups of chemical characterized and annotated metabolites and their related pathways, whereas in the untargeted analysis the study includes a comprehensive measurement of all metabolites present in samples [24,25].

The type of approach chosen will determine the experimental design, sample preparation, and which analytical techniques can be used to obtain the results. Both targeted and untargeted follow the similar pipeline. Briefly, the study design includes the population that will be part in the study and also the determination of the conditions that are relevant for the hypothesis in investigation, namely the sample size, randomization (as a study design consideration), storage (as a sample handling issue), freeze/thaw cycles and timing during sample preparation are the most common factors that should be taken into account to guarantee reproducible and successful experiments minimizing variability. There are three main analytical platforms frequently used in metabolomic studies, which include mass spectrometry (MS) and nuclear magnetic resonance (NMR) spectroscopy [26]. Moreover, after data acquisition, the obtained dataset, normally is subjected to statistical analysis (univariate and multivariate methods) to find significant variations that allow the discrimination of patients with a specific disease (in this case, BC) from a control group [27]. The most common approaches for the identification of important metabolites comprise the application of unsupervised methods, such as, principal component analysis (PCA), hierarchical cluster analysis (HCA), as well as supervised methods, like partial least squares discriminant analysis (PLS-DA), random forest (RF) and support vector machines (SVM) [26,28]. A training set is used to construct the multivariate analysis models (e.g., PCA or PLS-DA), followed by an external validation set to predict the new cohort of samples using the model constructed with the training model. Finally, the putative biomarkers can be placed in metabolic networks to allow the biological interpretation or which pathways are up- or down-regulated.

## 2. OMICS Science

The OMICs is a neologism broadly adopted in biomedical research, that comprises the dataset of genomics (DNA), transcriptomics (RNA), proteomics (proteins) and metabolomics (metabolites) based on the central dogma of molecular biology [29]. The purpose of OMICs science in cancer research is to discover cancer-specific biomarkers (diagnostic, prognostic and/or putative). The Food and Drug Administration (FDA) defined biomarkers as a “characteristic that is objectively measured and evaluated as an indicator of normal biological processes, pathogenic processes, or biological responses to a therapeutic intervention” [30]. Biomarkers are powerful tools, when used for the early cancer detection and selection of therapeutic strategy, thus improving the outcome of cancer treatment and reduce cancer-related mortalities.

One of the newest promising OMICs sciences is metabolomics being a suitable tool that provides state of the art of analytical instrumentation tandem with pattern recognition procedures and chemometric tools to discover new disease- biomarkers providing novel insights into disease etiology, and more robust assessment of etiological pathways [30,31]. Metabolomics studies the complex interaction in biological systems providing a comprehensive and detailed information of the phenotype and molecular physiology as result of environmental factors, genetic as well as exogenous and endogenous factors (e.g., age, gender, race, diet, drugs, exercise, gut microbiota) [31]. In addition, metabolomics can be used in early detection and diagnosis of cancer, in the assessment of therapies and medical interventions, since cancer is a disease that promotes changes in cellular metabolism [32,33,34,35]. This OMICs tool has been extensively applied in clinical health practice due to its ability to quickly analyze biological samples (e.g., blood, tissue, saliva and urine) with relatively simple sample preparation (10–30 min), cost-effective and high-throughput [30,36,37]. Nevertheless, metabolomics present several drawbacks resulting from biological and experimental features, such as sampling variability, inter- and intra-individual differences and a lack of validated protocols for biological samples handling which have a significant impact on the OMICs data approach [38,39].

The current review is focused in the metabolic profile of several biological samples, including lipidomics (lipids), labeled substrates (e.g., ^13^C labeled glucose), volatomic (volatile organic metabolites), and metabolites resulting from Krebs cycle [30,39] with the purpose of an early diagnosis, metabolic reprogramming, cancer typing, staging and therapeutic intervention response [29,37]. Regarding the Krebs/TCA cycle, there is evidence that the role of TCA for energy production and macromolecule synthesis by cancer cells, especially those with dysregulated oncogene and tumor suppressor expression [40,41,42]. Over the last years, there has been a rapidly growing number of metabolomic studies intended to discover new biomarkers or make disease diagnosis using different biological matrices, such as cell lines [43,44,45,46], blood [47], exhaled breath [48], plasma [33,49,50], saliva [51,52,53,54], tissues [55,56,57], serum [58] and urine [59]. In Table 1 are resumed the most common analytical approaches used in metabolomic studies grouped by type of biological sample and objective of the study. Interestingly, the main studies involve a diagnostic purpose using BC cell lines with the aim of search biomarkers, inspect the metabolome (endo- and exo-). Moreover, lipids as building blocks of cell membranes have their levels changed during the malignant transformation. Lipid metabolism plays a vital role in oxidative stress and is correlated with other parameters linked to BC risk (e.g., hormonal balance, body mass index, breast density, drug metabolism and growth of insulin levels) [43,60]. In addition, a summary of the total identified metabolites by analytical platform as well as the number of samples used for each biological specimen type is shown as Supplementary Materials (Figure S1).

In literature, the reports performed involving human cell lines focus mainly in diagnostic purpose. As for example in the volatile composition (VOMs) as described by Silva et al. [44] where the volatomic signature of BC cell lines was established, and based on the results, 2-pentanone, 2-heptanone, 3-methyl-3-buten-1-ol, ethyl acetate, ethyl propanoate and 2-methyl butanoate were detected only in cultured BC cell lines. These VOMs are formed endogenously or obtained from exogenous sources (e.g., environmental, lifestyle, biological agents) [51], and can be recognised as a useful tool to BC non-invasive diagnosis [44,51]. Other study by Willmann et al. [46] observed the changes of the exo- and endometabolite profiles in BC cell lines by LC-MS/MS and observed a clear discrimination of the breast epithelial from the BC cell lines through statistical tools. Moreover, a decrease on ratio of glutathione (GSH) and glutathione disulfide (GSSG) was observed in BC cell lines as a result of oxidative stress. The lipidomic profile of several BC cell lines was compared with normal cells obtained from non-cancerous tissues by LC-MS/MS and GC-MS that changes observed in breast tumor tissues were caused mainly by difference in lipidomic profiles of tumor cells and these alterations can be correlated with the lipidomic composition of the nine breast cancer cell lines. Furthermore, Martineau et al. [61], determined the absolute concentration of several metabolites (e.g., alanine, lactate, threonine, taurine, glutathione, glutamate, glutamine, choline, valine, isoleucine, myo inositol, serine, proline, aspartate and histidine), revealing the usefulness for the establishment of potential biomarkers. Also, BC cell lines with BRCA1 pathogenic mutations were investigated by LC-MS/MS in order to obtain their metabolic signature as possible diagnostic approach.

Regarding plasma, serum or blood, many studies have been conducted as observed in Table 1, with multiple aims as Cala et al. [49] that developed a pilot control case-study, where a metabolomic and lipidomic approach was performed in order to establish a plasma metabolic fingerprint of Colombian Hispanic women with BC. According to these authors, the plasma metabolites could contribute to an enhanced knowledge of the underlying metabolic shifts driven by BC in women of Colombian Hispanic origin. Moreover, despite racial differences, the mapped metabolic signatures in BC were comparable but not identical to those described for non-Hispanic women. Wang et al. [47] used a dried blood spot approach for rapid BC detection. In the first study, the target analytes were 23 amino acids and 26 acylcarnitines, and based on the results piperamide, asparagine, proline, tetradecenoylcarnitine/palmitoylcarnitine, phenylalanine/tyrosine, and glycine/alanine could be used as potential biomarkers to diagnose BC. Lyon et al. [70] established a serum metabolome analysis from the tryptophan pathway of 19 women with early-stage BC. The targeted analysis indicated higher kynurenine levels and kynurenine/tryptophan ratios post-chemotherapy. Also, the symptoms of pain and fatigue had association with several targeted metabolites. An improved metabolic profile of human serum samples was obtained using complementary thecniques, namely MS and NMR and this approach may be useful to achieve more accurate disease detection and gain more insights regarding disease mechanisms and biology [67].

Another study conducted by Lécuyer et al. [31] combined metabolomic and epidemiological approaches by NMR to investigate whether plasma untargeted metabolomic profiles could contribute to the identification of BC at-risk women, whereas Playdon et al. [72] focused on the evaluation of the associations of diet-related metabolites with the risk of breast cancer. It was possible to verify that the prediagnostic serum concentrations of metabolites related to alcohol, vitamin E, and animal fats were associated with ER^+^ breast cancer risk.

Urine became a very interesting biological sample to investigate as diagnostic tool or as result of a treatment, as it is easy to collect, and also as ending point of all reactions that occur in the body. Furthermore, Porto-Figueira [59] established the urinary volatomic biosignature from breast (BC), and colon (CC) cancer patients as well as healthy individuals. This last work observed that several pathways are over activated in cancer patients, being phenylalanine pathway in BC and limonene and pinene degradation pathway in CC the most relevant. Yu et al. [84] explored the relationship between urinary metabolites and clinical chemotherapy response in BC. As results, chemotherapy-sensitive patients exhibited 30% of change in metabolite levels when compared to healthy individuals, while chemotherapy-insensitive patients showed only 9% of change in metabolite levels when compared to healthy people that presented recurrence.

Another explored biological fluid is saliva as described by Zhong et al. [53] that screened the putative salivary biomarkers for BC diagnosis, staging, and biomarker discovery. As a result, 18 biomarkers were identified, but only three up-regulated metabolites, displayed the area under the curve (AUC) values higher than 0.920, indicating the high accuracy to predict BC. Also, Cavaco et al. [51] screened salivary volatiles for a putative BC discrimination, and from metabolites identified, only 3-methyl-pentanoic acid, 4-methyl-pentanoic acid, phenol, p-tert-butyl-phenol, acetic, propanoic, benzoic acids, 1,2-decanediol, 2-decanone, and decanal were statistically relevant for the discrimination of BC patients in the populations analyzed. Another type of molecules, the polyamines were associated with tumor growth due to their biosynthesis and accumulation [54]. In this context, Tsutsui et al. [52] and Takayama et al. [54] determined polyamines including N-acetylated forms in saliva to diagnose BC. According to Tsutsui et al. [52], the level of polyamines increased in BC patients, and the levels of *N*^1^-acetyl-spermine, *N*^1^*N*^8^-diacetyl-spermidine and *N*,*N*-diacetyl-spermine were significantly higher only in the relapsed patients. Takayama et al. [54] demonstrated that eight polyamines are strongly correlated with the BC patients. Furthermore, the ratio of *N*^8^-acetyl-spermidine/ (N^1^-acetylspermidine + *N*^8^-acetyl-spermidine) may be adopted as an index of the health status after the surgical treatment.

*In-vitro* analysis of BC tissues can be a valuable tool to inspect the metabolic differences between tissue classes, either using the hydrophilic or the lipophilic part. As a result, one might use the metabolomic profile as a novel tool for cancer characterization. Breast tissue is also an interesting biological sample used for diagnostic purposes and /or response to a treatment as demonstrated by Euceda et al. [79] that explored the effect of the antiangiogenic drug bevacizumab on metabolic profile from BC tissue. On the other hand, Budczies et al. [77] studied the glutamate enrichment as a new diagnostic opportunity in BC, and a positive correlation between glutamate and glutamine in normal breast tissues switched to negative correlation between glutamate and glutamine in BC tissues. Euceda et al. [79] observed a metabolic alteration indicating a decline in glucose consumption as an effect of chemotherapy. In addition, a lower glucose and higher lactate level was observed in patients (≥90% of tumor reduction) when compared to those with no response (≤10% of tumor reduction). In turn, Choi et al. [81] determined the metabolic profiling of core needle biopsy samples in order to predict pathologic response to neoadjuvant chemotherapy in patients with locally advanced BC. These authors observed that there was a trend of lower levels of phosphocholine/creatine ratio and choline-containing metabolite concentrations in the pathologic complete response group when compared to the non-pathologic complete response group. Most of the BC patients undergo a cycle or more of chemo being the general treatment that uses cancer-killing drugs before (neoadjuvant or preoperative therapy) and after (adjuvant therapy) surgery [31,36], Then, the therapeutic chemo effect may shift significantly between patients, as a result of BC phenotypes [37] of and intra- and inter- individual differences. For this reason, it is necessary to punctually and accurately evaluate the therapeutic effects of chemotherapy, which could help to adjust the chemotherapy regimen [71,84]. whereas the advances in treatment increased significantly the survival rates for women with BC, as women often report psychoneurologic symptoms (e.g., pain, fatigue, depression) during and after chemotherapy cycles. 

**Figure 3:**
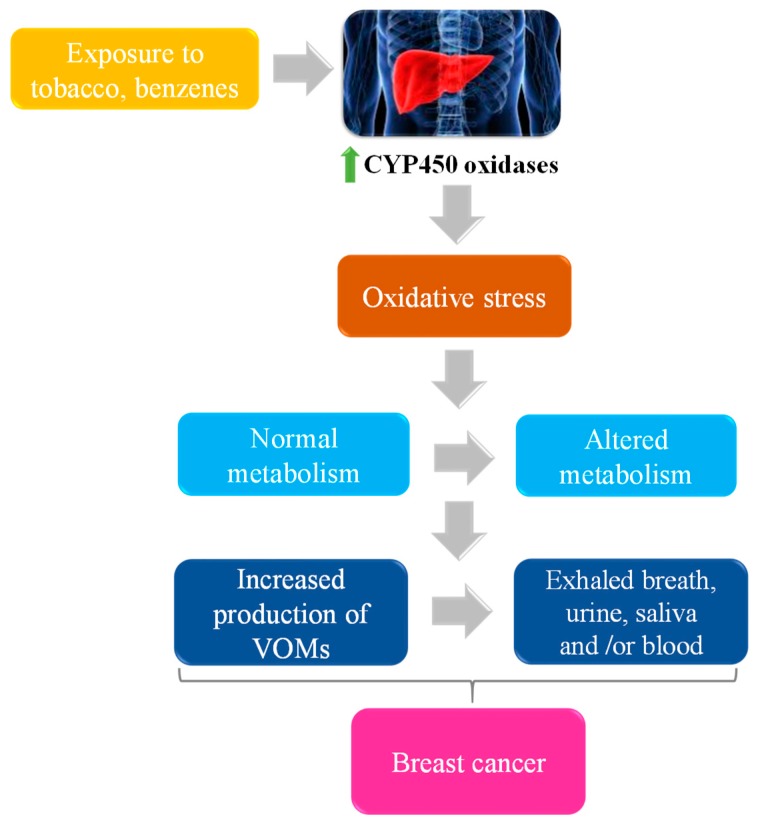
Schematic illustration of possible origin of some VOMs.

Regarding exhaled breath a less explored biological sample in terms of BC diagnostic purpose. In a study performed by Martinez-Lozano Sinues et al. [48] who developed a pilot study to identify cancer–related volatile profile in exhaled breath of BC patients. Concerning exhaled breath and the possible mechanisms involved in the production of endogenous VOMs, in Figure 3 is represented a schematic illustration about the possible pathways.

The principle behind this is based on the fact that the cancer growth is promoted by the progressive accumulation of genetic and epigenetic changes leading to cellular oxidative stress, which in turn increases the liver’s production of cytochrome P-450 (CYP450) oxidase enzymes to take into account with stress. Both processes affect the abundance of VOMs in breath once oxidative stress causes lipid peroxidation of polyunsaturated fatty acids (PUFA) in membranes, producing alkanes and methylalkanes which are catabolized by CYP450 [85].

## 3. Analytical Approaches

**Figure 4:**
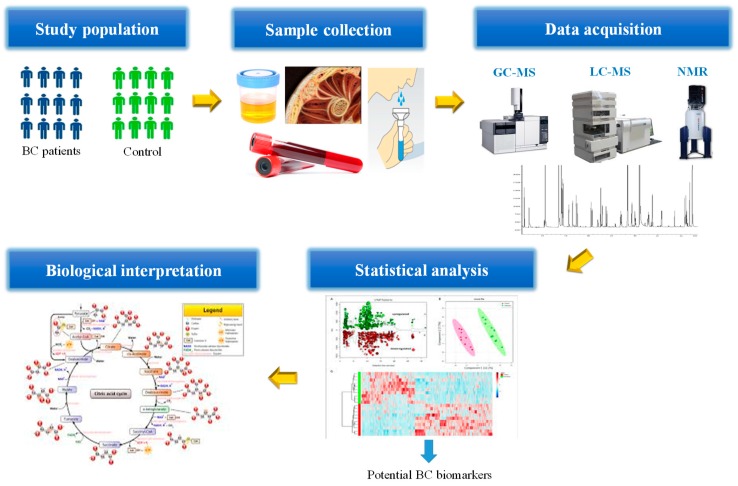
General flowchart in targeted/untargeted metabolomic approaches.

Metabolomics encompasses targeted and non-targeted analysis of endogenous and exogenous metabolites (<1500 Da), such as lipids, amino acids, hormonal steroids, peptides, nucleic acids, organic acids, vitamins, thiols and carbohydrates, which represent a promising tool for biomarker discovery [86,87]. The complexity of the metabolome, the metabolites properties and their concentration levels in biological samples complicates the separation and detection on a single analytical platform. For this fact, the integration of high resolution analytical frameworks, mass spectrometry (MS) and nuclear magnetic resonance (NMR), appear as an outcome in metabolomics studies, providing sensitive, reliable detection and quantification of thousands of metabolites in a biological sample and related metabolic pathways within a few minutes [27,86,87] as shown in Figure 4.

This review will provide an update of the most commonly used analytical methods in metabolomics, namely MS- and NMR- based metabolomics [27]. 

### 3.1. MS–Based Metabolomics

MS is an analytical tool extensively used in metabolomics applications, ranging from understanding the structural characterization of important metabolites to biomarker discovery [86]. Metabolic fingerprinting is general obtained by MS direct-injection, but this approach presents several drawbacks namely co-suppression and low ionization efficiency. Thus, generally MS based metabolomics includes a separation step, based on gas chromatography (GC–MS) [43,44,51,59,65,66,77,82], liquid chromatography (LC–MS) [33,43,46,50,52,53,54,55,70] or capillary electrophoresis (CE-MS) [83,84], to solve the co-suppression and to decrease the complexity of the biological sample. The integration of MS with a chromatographic technique (GC, LC) and capillary electrophoresis showed high sensitivity, speed, selectivity and improves the accuracy of compound identification, detection and quantification. In addition, GC-, LC- and CE-MS are destructive methods, requires sample preparation and are expensive, being these facts the main drawbacks of these hyphenated frameworks [86,88,89].

#### 3.1.1. Gas Chromatography-mass Spectrometry (GC-MS) - Based Metabolomics

In the last decades, MS and chromatography have been broadly developed, and GC-MS becomes a core and reliable separation, detection and identification analytical framework on metabolomic analysis [43,44,51,59,65,66,77,82]. After sample collection and metabolite extraction, a small volume of sample is commonly injected in splitless mode, once the metabolites are in trace levels, to improve the sensitivity and the carrier gas propels the sample through the high-resolution capillary column (30 or 60 m columns with 5–50% phenyl stationary phases). The separation in GC occurs in an oven at high temperatures, and the metabolites need to be thermally stable and volatile (e.g., aldehydes, ketones, alkanes, organic acids) or non-volatile metabolites requiring derivatization (e.g., amino acids, sugars, phosphorylated metabolites, amines, lipids) [86,88,89]. The samples are ionized by electron-impact (EI) or chemical ionization (CI) for MS detection, being EI the most used since it provides molecular ion fragmentation to obtain a mass spectrum revealing of the metabolite’s structure [88]. The MS employed influences the sensitivity of detection, being the quadrupole (q), time-of-flight (TOF) and ion trap the most usually applied in metabolomics. GC-qMS was used to screen salivary volatiles for putative BC as an exploratory study involving geographically distant populations [51], also to establish the metabolomic signature of human BC cell lines [44] and to discriminate different types of cancer based on urinary volatomic biosignature [59], among other examples reported in Table 1. In the first study, up to 120 volatiles from distinct chemical classes, with significant variations among the groups, were identified [51], whereas Silva et al. [44] and Porto-Figueira et al. [59] identified 60 and 130 volatiles in BC cell lines and urine, respectively. On the other hand, Budczies et al. [77,82] used GC-TOFMS framework to evaluate the glutamate enrichment as new diagnostic opportunity in BC and to accomplish a comparative metabolomics of estrogen receptor positive (ER^+^) and estrogen receptor negative (ER^−^) in BC. Budczies et al. [82] identified 19 metabolites BC tissues revealed significantly differences in central metabolism in ER^−^ when compared to the ER^+^ type. The affected metabolic pathways included the metabolism of glutamine with a decrease in concentration of glutamine and an increase glutamate and 2-hydroxyglutaric acid [82]. In turn, Dougan et al. [66] used GC-MS to evaluate the detectability, reliability, and distribution of metabolites measured in pre-diagnostic plasma samples in a pilot study of women listed in the Northern California site of the BC Family Registry. In this study. 661 metabolites were detected, 338 (51%) of them were found in all samples, and 490 (74%) in more than 80% of samples.

The main advantages of GC-MS-based metabolomics are sensitivity, specificity, high-throughput technology to handle a large volume of samples and reproducible. Nevertheless, this hyphenated technique has limited in mass range (*m*/*z* 30–550), the molecular ion is often not detected owing to fragmentation, which makes more difficult the identification of unknown metabolites and the metabolites need be volatile and thermally stable [89,90].

#### 3.1.2. Liquid Chromatography-Mass Spectrometry (LC-MS) - Based Metabolomics

Currently, liquid chromatography (LC) in particular high-performance liquid chromatography-mass spectrometry (HPLC-MS, LC-MS) represents an easy-going tool on separation and characterization of a metabolites pool, namely salts, acids, bases, hydrophilic and hydrophobic metabolites. The versatility of LC-MS is due to the several separation procedures and wide-ranging mass analyzers [90]. Contrarily to GC-MS, HPLC-MS is not limited to volatile and thermo stable metabolites and it is a promising tool for global metabolomics and the establishment of disease biomarkers.

Basically, the metabolites are eluted through a column based on their selective partition between a stationary phase (column material) and a mobile liquid phase. The metabolites according to the type of stationary phase can be eluted based on their charge, size, hydrophobicity and molecular weight [91]. Nowadays, the evolution of the HPLC is focused in miniaturization, smaller columns and low solvent volumes to attain a faster separation of metabolites. Ultra-high performance chromatography (UHPLC) appears as solution, since compared to HPLC promotes the resolution within a low analysis time and requires low volumes of solvent [92,93]. UHPLC columns are packed with 2 µm particles and the system operates at higher pressures (1000 bar) and tandem with MS, results in higher peak capacity, resolution, specificity and high-throughput abilities (reduced run time per sample) compared with HPLC [86,90,92,93,94].

Furthermore, Willmann et al. [46] analyzed the endo- and exometabolite of the BC cell lines MDA-MB-231, -453 and BT-474 as well as the breast epithelial cell line MCF-10A through two different analytical platforms: UHPLC-ESI-QTOF and HPLC-ESI-QqQ, which resulted in the identification of 92 annotated exometabolites and 58 endometabolites. In turn, Jové [33] used LC-ESI-QTOFMS/MS to establish the metabolomic profile of BC, whereas HPCL-ESI-MS was used to determine the determine the lipidomic differences between human BC and the surrounding normal tissues [55]. UHPLC tandem with MS was applied to explore novel blood plasma biomarkers associated to the BRCA1-mutated phenotype of BC [50], to determine polyamines including N-acetylated forms in saliva [52,54], and to screen the potential salivary biomarkers for BC diagnosis, staging, and biomarker discovery [53].

### 3.2. NMR–Based Metabolomics

NMR spectroscopy has been announced as a promising tool of metabolomics, providing a comprehensive view of metabolite fingerprinting, profiling and metabolic flux analysis under specific conditions, despite its inherent lower sensitivity compared to MS, limiting its skill with trace level metabolites. The main advantages of NMR are automation, requires low or no sample preparation, high reproducibility, non-destructive, non-selectivity in metabolite detection and the ability to simultaneously quantify multiple classes of metabolites [29,87].

The principle of NMR spectroscopy is based on the fact that the nucleic of many isotopes (e.g., ^1^H, ^13^C, ^14^N, ^15^N, ^17^O), when placed in a magnetic field, absorb radiation at a specific frequency [90]. The result is a NMR spectrum which corresponds to a unique metabolite pattern and provides structural information that can simplify the identification of unknown metabolites [86,89]. A fast identification of metabolite results from a combination of chemical shifts, spin–spin coupling, and relaxation or diffusion information [86,89]. Jobard et al. [68] reported a ^1^H NMR-based metabolic phenotyping study aiming the identification of metabolic serum changes associated with advanced metastatic BC (MBC) in comparison to the localized early disease (EBC). Histidine, acetoacetate, glycerol, pyruvate, glycoproteins (N-acetyl), mannose, glutamate and phenylalanine were the metabolites that allowed the discrimination between MBC and EBC groups. NMR was also used by Tenori et al. [58] to explore whether serum metabolomic spectra could distinguish between early and metastatic BC patients and predict disease relapse, whereas Singh et al. [63] used NMR to detect alterations in metabolites and their linkage to metabolic processes in a number of pathological conditions including BC. In the last study, the authors observed an increase in lipoprotein, lactate, lysine and alanine level and a decrease in the levels of pyruvate and glucose in serum of inositol 1, 4, 5-trisphosphate (IP3R) receptor group patients when compared to control. In addition, NMR offers the possibility to study tissue through high-resolution magic angle spinning (HR-MAS) to reduce line widths in NMR spectra of tissue samples [74,75,79,80,81]. Tayyari et al. [74] performed the metabolomic analysis of triple-negative and luminal A BC subtypes in African-American using HR-MAS-NMR. A total of 27 metabolites were assigned and the metabolic profiles of these subtypes were also distinct from those revealed in Caucasian women. In turn, the feasibility of HR-MAS-NMR of small tissue biopsies to distinguish between tumor and non-involved adjacent tissue was investigated by Bathen et al. [75]. The results showed that the levels of glucose were higher in samples with low tumor content, whereas samples with high tumor content presented higher levels of ascorbate, lactate, creatine, glycine, taurine and the choline-containing metabolites. Euceda et al. [79] evaluate the metabolomic changes during neoadjuvant chemotherapy combined with bevacizumab in BC using HR-MAS-NMR. According to these authors, despite metabolic profiles not being able to predict the pathological complete response (pCR) prior to treatment, a significant metabolic difference in pCR^+^ patients compared to pCR^−^ was detected after neoadjuvant chemotherapy.

### 3.3. Comprehensive Analytical Frameworks on Metabolomics Approach

Comprehensive analytical frameworks have gained popularity on metabolomics field [86], being hundreds of metabolites detected simultaneously through analytical frameworks such as GC×GC-MS, HPLC-CE-MS, LC×LC-MS, LC-MS-NMR, MALDI-FT-ICR-MS, LC-FT-ICR-MS, among others. In the last decade, two dimension (2D) liquid-liquid chromatography (LC×LC) as well as gas-gas chromatography (GC×GC) have been gained increasing attention since overcome overlapping of metabolites by diverting each peak from a GC or LC column to a second GC or LC column, improve sensitivity and complementary selectivity being a promising tool in metabolomics field [95]. Nevertheless, other comprehensive analytical framework has been purposed in metabolomic field, in this context LC-MS-NMR platform is used in the identification of unknown metabolites in biological samples at trace levels, providing sample efficiency higher than the conventional flow injection methods [86]. In this sense, Reichenbach and co-workers [96] developed a suitable approach based on GC×GC-HRMS to analyse a cohort of 18 samples from BC tumors. This approach avoided the intractable problem of comprehensive peak matching, through a few reliable peaks for alignment and peak-based retention-plane windows to define comprehensive features that can be consistently matched for cross-sample analysis. In addition, a clear discrimination was achieved between sample of different grades and establish potential BC biomarkers. On the other hand, Yu et al. [97] optimized GC×GC-MS for robust BC cells, tissue, serum and urine metabolite profiling. GC×GC-MS analysis revealed detection around 600 molecular features from which 165 were characterized representing different chemical groups, such as amino acids, fatty acids, lipids, carbohydrates, nucleosides and small polar components of glycolysis and the Krebs cycle using EI spectrum matching. NanoLC-FT-ICR MS was used to analyse protein digests of ~3000 laser capture microdissection (LCM)-derived tumor cells from breast carcinoma tissue, corresponding to ~300 ng of total protein [98].

## 4. Data Analysis

Data analysis is crucial in metabolomics, being indispensable in every step of research, namely in sampling and experiment designs, data pre-processing and metabolite identification, as well in variables selection, classification modeling and validation procedures. The great challenge of data analysis in metabolomics is the high dimensionality and complexity of datasets under analysis. Several chemometric tools and statistical softwares are used in order to attribute value for high-dimensional metabolomic information obtained previously by the analytical tools [99,100]. Normally, a complete data analysis procedure in metabolomics is based on the following steps: dataset pre-treatment (centering, scaling, normalization), pre-processing (exploratory projection, variables selection), processing (predictive models), validation (model verification) and post-processing (pathway analysis) [101]. However, data analysis is dependent on the objective of the study and may be a simple exploratory research or complex discovery of biomarkers and metabolic pathways, for this reason not all steps are always present or are not followed in the same order. The data analysis procedures of recent metabolomics studies in BC are described in Table 2.

### 4.1. Dataset Pre-Treatment

Dataset pre-treatment is the initial step in data analysis, being extensively used in metabolomics to resolve the heteroscedasticity of high-dimensional datasets. Commonly, pre-treatment in BC metabolomics is done through normalization of dataset based on the centering, scaling, transformation and/or experimental corrections of variables values [103,104,105]. Centering is performed when the data analysis is focused on the differences between variables, where all measurements (e.g., concentrations, areas) are converted to values around zero based on variation measures. Mean [46,67,68,79] is the measure normally used in centering. Scaling is used to adjust the variables measurements based on a scaling factor, converting the measurements of all variables into values relative to the scaling factor. The scaling factor selected can be a dispersive measure (e.g., standard deviation) or size measure (e.g., mean). The main scaling approaches based on dispersive measures are autoscaling (standard deviation) [46,51,59] and pareto scaling (square root of the standard deviation) [43,53,55]. On the other hand, the most of size measure approaches uses scaling factors based on the mean [80], median [51,57,59,66,75,78,83] or total intensity value [53,58,67,68,71,73,74,81]. Transformations are mathematical approaches used to decrease the heteroscedasticity of dataset, which the variability between variables is dramatically reduce. Log [43,57,66,69,70,72,76,79] is the main transformation in BC metabolomics. However, cubic root [51,59] and quantile [48] transformations are also used. Other normalization approaches based on experimental corrections are also used in metabolomics, such as internal standards [102,106] and sample weight [61]. Internal standards normalization assumes that the heteroscedasticity of all variables is systematic and can be corrected by variance of internal standards. Sample weight normalization is the direct correction of variables values by experimental sample measures (e.g., volume and weight).

### 4.2. Pre-Processing

Pre-processing methods are performed to obtain an exploratory projection of dataset or an overview of variables importance prior to prediction models processing. Primarily, normality tests are used to determine if the data distribution is normal (parametric) or not normal (non-parametric). The most commonly used are Kolmogorov-Smirnov test (KS-test) [50,73], Shapiro-Wilk test (SW-test) [73] and Lilliefors test (L-test) [73]. Two types of approaches are normally used in exploratory projections/variables importance ranking of BC metabolomics datasets: univariate and multivariate analysis. Univariate statistical methods are used to analyzed only one variable at a time, being useful to easily discover significant differences or measure correlations between samples groups. The differentiation is based on variance between groups by rejection of the null hypothesis or acceptation the alternate hypothesis [101,107,108]. The most common methods used when the data is parametric are T-tests [31,47,48,50,53,57,59,62,68,70,71,73,74,76,77,78,79,84] and ANOVA [33,44,46,63,64,66,68,69,72,83] T-tests, such as Student and Welch’s tests, are recommended to analyze differences between two groups, and ANOVA-based methods, such as one-way ANOVA, two-way ANOVA, factorial ANOVA and MANOVA are used to evaluate more than two groups. Alternative univariate methods are implemented when the assumption of the normal distribution is non-parametric, such as Mann-Whitney test (MW-test) [51,81,102] and Wilcoxon test (W-test) [58]. In addition, univariate methods are also widely used to measure the correlations between continuous variables and response. The Pearson correlation [46,70,72,76,77,79] is the preferred option for linear relationships in populations with normal distribution. On the other hand, the Spearman correlation [31,80,82] is usually used in non-parametric datasets. More complex correlation methods are also used in data analysis, such as Correlation Feature Selection (CFS) [65], where the appropriate correlation measure and a heuristic search strategy are performed by experiments on artificial and natural datasets based on algorithms.

Similarly, the multivariate methods are also widely used for exploratory studies to obtain dataset patterns based on relationships between groups, being divided into two sub-groups, unsupervised and supervised methods. Unsupervised methods are the preferential option for exploratory studies, where the modeling process is based only on the explanatory variables, without external intervention of user (Yi et al., 2016). The most commons are principal component analysis (PCA) [56,57,60,62,70,75,76,80,92,93] and hierarchical cluster analysis (HCA) [33,43,51,56,59,76] PCA provides the projection of dataset into low dimensional based on orthogonal transformation, converting the variables variability from a set of observations into score vectors and loadings, called principal components [100,109]. HCA methods are used to form subsets of samples at ordered levels based on variables similarities/dissimilarities (such as distances or correlations) and can be performed in agglomerative mode (samples are aggregate into clusters) or divisive mode (complete dataset is divided into clusters). In both modes, the linkage criterion need to be selected, being that the most commonly used are single-linkage clustering (the minimum of distances) and complete linkage clustering (the maximum of distances) [110,111].

### 4.3. Processing Methods

After the explorative studies and variable selection, the next step is the processing of dataset in order to create a predictive response model to classification of new samples (ex. diagnostic tools), identification of valuable variables (ex. biomarkers) or exploring the mechanisms of metabolomic studies (ex. metabolic pathways). In this stage, the supervised methods are the preferential choice, where the response models are mainly based on two types, continuous (regression) and discrete (classification) [100,103]. The main methods for continuous response are based on multiple linear regression (MLR), sometimes called ordinary least squares (OLS). MLR is performed to predict the values of a dependent variable (response) based on a set of continuous explanatory variables, assuming a linear combination of the explanatory variables [109]. The most applied MLR-based method in metabolomics is partial least squares (PLS) [44,55,68,71]. Unlike PCA, which uses only the variables variation, PLS is a predictive and supervised method that use an informative response to maximize the covariance between the explanatory variables and the response, producing score vectors and loading vectors. The prediction model is based on interaction between the variables and response, ignoring the variables with irrelevant importance. The importance of each variable is defined according the PLS-based criteria, such as loading weights, variable importance on projection scores, regression coefficient, target projection and selectivity ratio [100,101,109]. However, when categorical variables are introduced, the discrete models should be used. Discrete models provide a predictive classification of response based on continuous and categorical variables, being classified into linear or non-linear. In linear methods, the classification is performed by highest probability based on linear relationships between explanatory variables, where exist a grouping variable (categorical). Linear discriminant analysis (LDA) [44,54] is the preferential method to classification models of discrete responses. LDA perform linear transformations of explanatory variables to create discriminant functions that will maximize the separation between multiple classes of samples (groups) based on the information of the categorical variables [109]. Among the various LDA-based methods, PLS-DA [33,46,47,51,53,59,63,64,67,69,71,74,75,77,79,84,102] is most widely used in metabolomics studies. PLS-DA is a successful combination of PLS and LDA that provides a visual low-dimensional pattern of samples discrimination based on the analysis of relationships between continuous and categorical variables [101,109]. Recently, some extensions of PLS-DA were used in BC metabolomics, namely the OPLS-DA [43,51,67,73,81]. OPLS-DA separates out response orthogonal variations in rotations of the original component [109].

On the other hand, non-linear methods are used when metabolomics dataset follow a non-linear response. The most applied non-linear methods are support vector machines (SVM) [48,59,65,102], random forests (RF) [33,48,58,59,65,80] and logistic regression analysis (LRA) [31,47,65,69,72,102]. SVM is a kernel-based model used for regression and classification of non-linear datasets, transforming the non-linear data into more general spaces (linear) by algorithm based on kernels functions. SVM perform the mapping of dataset into a high-dimensional space through kernels functions for the separation of two groups of samples into distinctive regions. The separation is based on support vectors, which are points (samples) on the boundary or on the incorrect side of the margin supporting the separation. SVM is a versatile method that transforms non-linear complex datasets into a high-dimensional space where classes are linearly separable [100,101,109]. RF is a non-linear method for regression and classification of high-dimensional datasets, where a large number of classification and regression trees are created by bootstrapping (replacement) based on random selection of a training samples from the original dataset. Afterwards, bootstrapping is performed systematically to build a large group of simple trees that are used to estimate classification accuracy of the model [100,101]. Another non-linear predictive method widely used is LRA, which is similar to linear regression, but with a binomial response variable. LRA is used to explain the relationship between one dependent binary variable and one or more nominal, ordinal, interval or ratio-level independent variables [112].

### 4.4. Model Validation

The validation of predictive models is a key step in data analysis of metabolomics studies. Validation process analyzes the performance/ability of model to predict correctly the hypothesized relationships between variables and responses [101]. Several validation methods have been used in BC metabolomics. The coefficient of determination (R^2^) is the simplest method to evaluate the ability of predictive model, being used for continuous responses. The R^2^ is expressed as the ratio between 0 and 1, where a value of 1 indicates the perfect prediction. However, this validation is recommended for small datasets, due to fact that the R^2^ value tends to be increased when a predictor variable is added to the model [113]. However, in validation of predictive models used to high-dimensional and complex datasets, as the case of metabolomics studies, the cross validation (CV) methods are the preferential option. CV provides qualitative and quantitative analysis of the model ability to model’s ability to predict new independent samples without collecting additional data. During the CV, the available data are split into two sets, where one set is used to create a predictive model using the values of continuous and predictor variables (training set). The second set is used to test the performance of predictive model (validation set) [100]. The most applied CV procedure is k-fold (K-CV) [33,44,54,55,63,64,65,67,68,69,73,77,79,80,84,102]. K-CV processing is based on random partition of original dataset into equal sized subsamples (k). A single k subsample is used as the validation set for testing the model, and the remaining k -1 subsamples are used as a training set. This process is then repeated k times (folds), with each of the k subsamples being used exactly one time as the validation set [106]. One special type of K-CV is the leave-one-out cross validation (LOOCV) (Bathen et al., 2013; Choi et al., 2013; Cífková et al., 2017; Martinez-Lozano Sinues et al., 2015; Tayyari et al., 2018; R. Vettukattil et al., 2013; Willmann et al., 2016), where the number of folds equals the number of k subsamples. LOOCV is considered an exhaustive CV, being recommend for small datasets [106,113]. Another type of CV is the Monte Carlo cross validation (MCCV) [51,59]. Although less used in metabolomics than LOOCV, MCCV is asymptotically consistent and showed better prediction ability. In MCCV proceeding, significant part of dataset is leaved out at a time during model validation, repeating systematically this procedure several times [114,115]. The Q^2^ value, which is the equivalent R^2^ value, is the preferential coefficient of determination for CV procedures. 

A visual and easy model validation method is the receiver operating characteristic (ROC) curve [31,33,43,47,48,53,54,58,59,63,65,67,68,69,71,73,74,77,102] which the prediction ability of a model is validated considering the specificity (ratio of the correctly predicted negatives) and sensitivity (ratio of correctly predicted positives). The ROC curve is given by plotting the sensitivity versus (1 - specificity) across a series of cutoff points. The area under curve (AUC) is a quantitative measure (between 0 and 1) of the ability of predictive model, where a AUC value close to 1 indicates a nearly perfect prediction response [100,113].

Random resampling-based methods are a robust alternative for model validation. The most used in BC metabolomics are bootstrapping [48,58,68,80] and permutation tests [47,51,53,79,80]. Bootstrapping is a model validation based on replacement of samples, which can be considered non-parametric when the replacement is from the original dataset, or parametric when random noise is added from a recognized distribution to the dataset to estimate underlying sampling distribution or establish robust confidence intervals. Normally, in metabolomics studies the common approach is non-parametric bootstrapping [113,116]. Permutation tests provide the exact control of false positives from a predictive model (linear or non-linear), under minimal assumptions, based on differences between the randomly permuted response variables model and the original model. Permutation tests are based on a repeatedly permuting (repetitive reordering) of the N entries in the response variable. Permuted vectors containing integers between 1 and N are produced in a random number generator, creating new scrambled response variables only by switching their internal positions. The scrambled vectors are modelled one by one, where for every test, the R^2^ and Q^2^ values are calculated and saved. After, these values are compared with the values calculated from the original data. The results of permutation tests are displayed as a percentage overlap between the real and permuted R^2^ and Q^2^ values, where a 0% of overlap is the optimal result [109,117].

### 4.5. Post-Processing

The post-processing step consists in the interpretation of metabolomic responses from original dataset. Normally, pathway analysis is the most used strategy to provide an overview of association/relationship between identified metabolites and metabolic pathways and other general biological networks. Pathifier [65] and metaboanalyst [33,59,63,73,74] are the most used software for this propose in metabolomics.

## 5. Future Directions

The advances in analytical techniques and chemometric methods in metabolomics have been growing rapidly becoming possible the identification of potential biomarkers. Furthermore, the integration of analytical platforms increases the comprehensive analysis of metabolites in biological samples. In this context, metabolites became valuable identifications, regardless their hierarchical source, enabling the phenotypic properties in a biological system. Additionally, the identification of key metabolic pathways from which significant metabolites are linked, it is possible to reveal potential targets for cancer therapy.

Also, standard procedures for sample collection, data analysis and shared in repositories have potential to be adopted by both researchers and medical communities.

Since the metabolome instantly responds to environmental stimuli including therapeutic or surgical intervention, could be also used to monitor the metabolic status of the individual and indicate any possible toxic effects. Moreover, metabolomics may help in the detection of potential cancer biomarkers, being useful for example in the development of different devices, including biosensors, that can significantly improve the cancer diagnosis. These devices include a biorecognition element within a biosensor system. The biorecognition molecules interact with the target, which is then converted into a measurable signal by a transducer. Basically, these molecules, usually enzymes or antibodies, can be immobilized on the transducer surface and interact with the target (biomarker) to produce a signal is interpreted, providing information about the disease and their possible recurrence after therapy.

## Figures and Tables

**Table 1:** Summary of metabolomics studies performed in breast cancer biomarker discovery in different biological matrices.

Biological Sample	Sample Groups	Aim	Analytical Approaches	Main Conclusions	References
Human cell lines	-	-	-	-	-
Diagnostic biomarkers	BC (ZR-75-1, T-74D, MCF7, MDA-MB-231, MDA-MB-453, MDA-MB-468, SK-BR-3, BT-474, BT-549), Control (MCF10A)	To compare the differences in the lipidomic compositions of human cell lines derived from normal and BC tissues, and tumor vs. normal tissues obtained after the surgery of BC patients.	LC-MS/MS, GC-MS	* 123 lipids were identified, and a differentiation was observed for MDA cells	[29,43]
Diagnostic biomarkers	BC (MDA-MB-231, -453, BT-474), Control (MCF-10A)	To determine endo- and exo-metabolite analysis of the BC cell lines	UPLC-MS/MS, LC-MS/MS	* Statistical analysis allowed a discrimination of the breast epithelial cells from the BC cell lines * MDA-MB-231 showed an increase in nicotinamide levels, namely in 1-ribosyl-nicotinamide and NADþ	[46]
Diagnostic biomarkers	BC (T-47D, MDA-MB-231, MCF-7), Control (HMEC)	To establish the BC cell lines volatile metabolomic signature	GC–MS	* 60 VOMs were identified and six of them were detected only in the headspace of cancer cell lines	[44]
Diagnostic biomarkers	BC (MDA-MB-468, SKBR3, MCF-7)	To quantify specific metabolites in BC cell extracts	NMR	* Significantly differences were observed between cell lines, namely in the concentrations of 15 metabolites * The current method represented a useful tool for the establishment of potential biomarkers	[61]
Diagnostic biomarkers	BC (Cal 51, SKBR3, MCF-7)	To measure the absolute metabolite concentrations in complex mixtures with a high precision in a reasonable time	NMR	* The proposed approach represented a powerful tool to quantify 14 metabolites (alanine, lactate, leucine, threonine, taurine, glutathione, glutamate, glutamine, choline, valine, isoleucine, myo-inositol, proline, and glucose) in cell extracts within 20 min	[45]
Diagnostic biomarkers	BC cell lines (MCF-7, HCC70, MDA-MB-231, MDA-MB-436, MDA-MB-468), BC patients (*n* = 35)	To investigate the metabolic profiles of human BC cell lines carrying BRCA1 pathogenic mutations	LC-MS/MS	* It was possible to collect differential metabolic signature for BC cells based on the BRCA1 functionality	[50]
Therapy response	BC cell line (MCF-7)	To develop a robust and highly sensitive platform to identify endogenous estrones in clinical specimens	MALDI-MS, LC-MS/MS	* The results suggested that MALDI-MS-based quantitative approach can be a broad method for the ketone-containing metabolites target analysis thus replicating the clinical stage.	[62]
Therapy response	BC tissue (*n* = 40), Blood (*n* = 27), BC cell lines (*n* = 3)	To detect alterations in metabolites and their linkage to metabolic processes in several pathological conditions including BC	NMR	* Functional of IP3Rs in causing metabolic disruption was observed in MCF-7 and MDA MB-231 cells * The results offered new insights regarding the relationship of BC metabolites with IP3R.	[63]
Metabolic reprogramming	MDA-MB-231, BC xenografts	To study toxic effects of bisphenol and the underlying mechanisms on tumor metastasis-related tissues	LC-MS/MS, MALDI-MS	* Metabolites-based studies might be suitable for BC diagnosis * The data provided good indication for BPA screening secure option	[64]
Human Blood, plasma, serum	-	-	-	-	-
Diagnostic biomarkers	BC patients (*n* = 258), Benign mammary gland (*n* = 159), Control (*n* = 78)	To screen metabolite markers with BC diagnosis potentials	MS	* The method developed allowed the discrimination of BC from non-BC using six blood metabolites	[47]
Diagnostic biomarkers	Metastatic BC patients (*n* = 95), Early-stage BC patients (*n* = 80)	To explore whether serum metabolomic spectra could distinguish between early and metastatic BC patients and predict disease relapse	NMR	* Disease relapse was linked with lower and higher levels of histidine and glucose, respectively	[58]
Diagnostic biomarkers	BC patients (n= 132), Control (*n*= 76)	To develop a new computational method using personalized pathway dysregulation scores for disease diagnosis	LC-TOF-MS, GC-TOF-MS	* The method allowed to determine important metabolic pathways signature for BC diagnosis, representing a suitable tool for diagnostic and therapeutic interventions.	[65]
Diagnostic biomarkers	BC patients (*n* = 45), Control (*n* = 45)	To detect differences between BC and healthy individuals	UHPLC-MS, GC-MS	* 661 metabolites were detected, but only 338 metabolites were found in all samples, and 490 in more than 80% of samples.	[66]
Diagnostic biomarkers	BC patients (*n* = 29), Control (*n* = 29)	To establish a plasma metabolic fingerprint of Colombian Hispanic women with BC	LC-MS, GC-MS, NMR	* The current report showed the effectiveness of multiplatform strategies in metabolic/lipid fingerprinting works	[49]
Diagnostic biomarkers	BC patients (*n* = 91), Control (*n* = 20)	To explore whether serum metabolomic profile can discriminate the presence of human BC irrespective of the cancer subtype	LC-MS/MS	* From the 1269 metabolites identified in plasma from controls and patients; only 35 metabolites were related to BC.	[33]
Diagnostic biomarkers	BC patients (*n* = 27), control (*n* = 30)	To apply 1H NMR and DART-MS for the metabolomics analysis of serum samples from BC patients and healthy controls.	NMR, DART-MS	* The approach allowed the disease classification and the biochemical validation useful to identify the mechanisms associated to BC development.	[67]
Diagnostic biomarkers	Metastatic BC patients (*n* = 39 + 51 for validation), Early-stage BC patients (*n* = 85 + 112 for validation)	To distinguish between early and metastatic BC	NMR	* Metabolic phenotyping by NMR showed a robust potential for the diagnosis, prognosis, and management of BC cancer patients	[68]
Diagnostic biomarkers	BC patients (*n* = 40) BE patients (*n* = 40) and healthy controls (*n* = 34). BE patients with fibroma (*n* = 25) and chronic fibroadenosis of breast (*n* = 15)	To investigate the free fatty acid (FFA) metabolic profiles to identify biomarkers that can be used to distinguish patients with BC (BC) from benign (BE) patients or healthy controls.	GC-MS	The FFA biomarkers proved to be helpful for the prevention and characterization of BC patients.	[69]
Therapy response	BC patients (*n* = 19)	To compare metabolite concentrations and Pearson’s correlation coefficients to examine concomitant changes in metabolite concentrations and psychoneurologic symptoms before and after chemotherapy.	UPLC-MS/MS	* The post-chemotherapy global metabolites were characterized by higher and lower amounts of acetyl-L-alanine and indoxyl sulfate and 5-oxo-L-proline, respectively. * Metabolomics was useful for further understanding of biological mechanisms associated with psychoneurologic symptoms.	[70]
Therapy response	BC patients (*n* = 28)	To identify potential biomarker candidates that can predict response to neoadjuvant chemotherapy for BC	LC-MS, NMR	* The concentrations of threonine, isoleucine, glutamine, linolenic acid had significantly different responses to chemotherapy * The purposed approach clearly discriminates patients regarding the response to drugs providing a valuable tool for a non-invasive prognosis of the treatment strategy.	[71]
Endogenous factors	BC patients (*n* = 206), Control (*n* = 396)	To investigate whether plasma untargeted metabolomic profiles could contribute to predict the risk of developing BC	NMR	* The study contributed to the development of screening approaches for the identification of BC at-risk women.	[31]
Endogenous factors	BC patients (*n* = 621), Control (*n* = 621)	To evaluate associations of diet-related metabolites with the risk of BC in the prostate, lung, colorectal and ovarian cancer screening trial	GC-MS, LC-MS/MS	* The data obtained showed how nutritional metabolomics might identify diet-related exposures associated to cancer risk.	[72]
Human urine	-	-	-	-	-
Diagnostic biomarkers	BC patients (*n* = 30), CC (*n* = 30), Control (*n* = 30)	To discriminate different types of cancer based on urinary volatomic biosignature	GC-MS	* The butanoate metabolism was highly activated in studied cancers, as well as tyrosine metabolism, but in a reduced proportion * Different clusters allowed to establish sets of VOMs fingerprints resulted in the discrimination of the studied cancers	[59]
Therapy response	BC patients (*n* = 31), Control (*n* = 29)	To identify metabolites which can be helpful in the understanding of metabolic alterations driven by BC as well as their potential usage as biomarkers	LC-MS, GC-MS	* The analytical multiplatform approach enabled a wide coverage of urine metabolites revealing significant alterations in BC samples	[73]
Human Saliva	-	-	-	-	-
Diagnostic biomarkers	BC patients (primary, *n* = 8; relapse, *n* = 22), Control (*n* = 14)	To determine polyamines including N-acetylated forms in human saliva and the diagnostic approach to BC Patients	UPLC−MS/MS	* The increase on polyamines level in BC patients Ac-SPM, DAc-SPD, and DAc-SPM levels were significantly higher only in the relapsed patients	[52]
Diagnostic biomarkers	BC patients (*n* = 30), Control (*n* = 25)	To screen the potential salivary biomarkers for BC diagnosis, staging, and biomarker discovery.	UPLC-MS	* Saliva metabonomics approach may provide new insights into the discovery of BC diagnostic biomarkers.	[53]
Diagnostic biomarkers	BC patients (*n* = 111), Control (*n* = 61)	To determine of polyamines including their acetylated structures for the diagnosis of BC patients.	UPLC-MS/MS	* The ratio of N8-Ac-SPD/ (N1-Ac-SPD + N8-Ac-SPD) can be used as a health status index after the surgical treatment.	[54]
Diagnostic biomarkers	BC patients (*n* = 66), Control (*n* = 40)	To explore the potential of the volatile composition of saliva samples as biosignatures for BC non-invasive diagnosis	GC-MS	* This study defined an experimental layout appropriate for the characterization of volatile fingerprints from saliva as potential biosignatures for BC non-invasive diagnosis.	[51]
Human Exhaled breath	-	-	-	-	-
Diagnostic tool	BC patients (*n* = 14), Control (*n* = 11)	To detect and identify human exhaled BC–related volatile profile	MS	* Eight metabolites enabled a clear discrimination of exhaled breath of BC patients from controls. * The analytical technique provided a non-invasive strategy to detect VOMs for the BC diagnosis.	[48]
Human Tissues	-	-	-	-	-
Diagnostic biomarkers	BC patients (*n* = 10)	To establish a detailed lipidomic characterization with the goal to find the statistically differences between BC and normal tissues.	HPLC-MS	* Total concentrations for phosphatidylinositols, phosphatidylcholines, phosphatidylethanolamines and lysophosphatidylcholines were increased leading to a clear differentiation by PCA and OPLS-DA.	[55]
Diagnostic biomarkers	Paired tumor and non-tumor liver (*n* = 60), breast (*n* = 130) and pancreatic (*n* = 76)	To assess the metabolomic profiling as a novel tool for multiclass cancer characterization	GC-MS, LC-MS	* The findings provided a framework to validate cancer-type specific metabolite levels in tumor tissues.	[56]
Diagnostic biomarkers	BC patients (*n* = 37), Control (*n* = 35)	To identify potential biomarkers that differs TNBC from ER^+^ BC	GC-MS, LC-MS/MS	* 133 metabolites presented significant differences between ER^+^ and TNBC tumors * The metabolic pathway of tumors can provide new treatment targets.	[57]
Diagnostic biomarkers	BC patients (*n* = 47), Control (*n* = 35)	To identify how TNBC differs from LABC subtypes within the African-American and Caucasian BC patients	HR-MAS-NMR	* Increased pyrimidine synthesis was related to TNBC in Caucasian women * Novel treatment targets for TNBC could be explored through the metabolic changes	[74]
Diagnostic biomarkers	BC patients (*n* = 228)	To distinguish between tumor and non-involved adjacent tissue	HR-MAS-NMR	* Metabolic profiling of tumor tissues by NMR can be a suitable method for the analysis of the resection margins during BC surgery	[75]
Diagnostic biomarkers	BC patients (*n* = 25), Control (*n* = 5)	To establish metabolic profiles of ER^+^ vs. ER^−^ and of ER^−^ subtypes linked to genetics	GC-MS, LC-MS	* Changes in the metabolic profile of ER^−^ vs. ER ^+^ breast tumors were observed * The data represents a potential tool for the hypothesis testing of tumor metabolism	[76]
Diagnostic biomarkers	BC patients (*n* = 270), Control (*n* = 97)	To quantify the dysregulation of the glutamate-glutamine equilibrium in BC	GC-TOFMS	* A positive correlation between glutamate and glutamine in normal breast tissues was observed, whereas a negative correlation was obtained for normal tissues	[77]
Diagnostic biomarkers	95 OC (84 peritoneal, 11 pleural), 10 BC (7 pleural, 2 peritoneal, 1 pericardial), and 10 malignant mesotheliomas (6 peritoneal, 4 pleural)	To identify the metabolic differences between ovarian serous carcinoma effusions obtained pre- and post-chemotherapy and compare ovarian carcinoma (OC) effusions with breast carcinoma and malignant mesothelioma specimens.	1H-NMR	* Differences in metabolic profiles of different malignant effusions were detected * Metabolic characterization by NMR can be a technique to additional knowledge the mechanisms of effusion development	[78]
Therapy response	BC patients (*n* = 122)	To explore the effect of neoadjuvant therapy on metabolic profiles of BC tissues	HR-MAS-NMR	* Non-metastatic breast tumor tissue reflected different alterations in all patient groups after treatment. * Metabolic profiles discriminated pNRs from pMRD patients thus complementing other molecular assays allowing the knowledge of the underlying mechanisms affecting the response.	[79]
Therapy response	BC patients (*n* = 18)	To study metabolite levels in human BC tissue, assessing, for instance, correlations with prognostic factors, survival outcome or therapeutic response	HR-MAS-NMR	* Significant changes between the tumors were identified, indicating that the intertumoral changes for numerous metabolites were greater than the intratumoral changes for these three tumors.	[80]
Therapy response	BC patients (*n* = 37)	To determine whether metabolic profiling of core needle biopsy (CNB) samples using HR-MAS-NMR could be used for predicting pathologic response to neoadjuvant chemotherapy (NAC) in patients with locally advanced BC	HR-MAS-NMR	* The purposed method can be applied to predict the pathologic response before neoadjuvant chemotherapy	[81]
Therapy response	BC patients (*n* = 271)	To establish metabolic signatures for ER^+^ vs. ER^−^ BC	GC-TOFMS	Some metabolites levels were increased in ER^−^ subtype, such as, beta-alanine, glutamate and xanthine The down-regulation of the ABAT protein in ER^−^ BC was confirmed by immunohistological analysis.	[82]
Mouse BC tissue	-	-	-	-	-
Metabolic reprogramming	MMTVPyMT, MMTV-PyMT-DB, MMTV-Wnt1, MMTV-Her2/neu, and C3(1)-SV40 T-antigen (C3-TAg)	To identify global metabolic profiles of breast tumors isolated from multiple transgenic mouse models and to identify unique metabolic signatures driven by these oncogenes	GC-MS, LC-MS/MS, CE-MS	* C3-TAg was the only cohort with a tumor metabolic signature composed of ten metabolites with significance prognostic value in BC patients	[83]

ANOVA – Analysis of variance; AUC – Area under the curve; BC – BC; BFS– Bootstrap feature selection; CE-MS – Capillary electrophorese-mass spectrometer; DART-MS – Direct analysis in real time mass spectrometry; GC-MS – gas chromatography – mass spectrometry; GC-TOF-MS – Gas chromatography time-of-flight mass spectrometry; GGM – Gaussian graphical modelling; HCA – Hierarchical cluster analysis; HR-MAS-NMR - High resolution magic angle spinning nuclear magnetic resonance spectroscopy; LC-MS/MS – Liquid chromatography tandem with mass spectrometer; LC-TOF-MS – Liquid chromatography time-of-flight mass spectrometry; LDA – Linear discriminant analysis; MALDI-MS – Matrix-assisted laser desorption/ionization mass spectrometry; MCCV – Monte Carlo cross validation; MS – Mass spectrometry; MWT – Mann Whitney U test; NMR – Nuclear resonance magnetic; NRI – Net reclassification improvement; OPLS-DA – Orthogonal projections to latent structures discriminant analysis; OSC-PLS – Orthogonal signal correction partial least squares; PC – Pearson correlation; PCA – Principal component analysis; PEA – Pathway enrichment analysis; PLS-DA – Partial least squares discriminant analysis; RF – Random Forest classifier; ROC – Receiver operating characteristic; SCC – Spearman correlation coefficient; SVM – Support vector machine; TNBC – Triple negative BC; UPLC-MS/MS – Ultra performance liquid chromatography tandem mass spectrometer; VIP – variable importance in projection; VOMs – volatile organic metabolites.

**Table 2:** Summary of the main chemometric methods applied to metabolomic studies.

Data Analysis						
Biological Sample	Data Pre-Treatment	Pre-Processing	Processing	Validation	Post-Processing	Reference
Diagnostic tool	-	-	-	-	-	-
Human BC cell lines	Scaling (Pareto scaled), Transformation (log transformed)	PCA, HCA	OPLS-DA	LOOCV, ROC	none	[43]
	Centering (mean centered), Scaling (autoscaled)	ANOVA, PCA, HCA, Pearson correlation	PLS-DA	LOOCV	none	[46]
	Experimental correction (sample weight corrected)	PCA	none	none	none	[61]
	none	none	none	none	none	[45]
	none	ANOVA, PCA	PLS, LDA	K-CV	none	[44]
Human blood	none	T-test	PLS-DA, LRA	ROC, Permutation test	none	[47]
	Scaling (total intensity value scaled)	Wilcoxon test	RF	ROC, Bootstrapping	none	[58]
Human Exhaled breath	Transformation (quantile transformed)	T-test	RF, SVM	LOOCV, ROC, Bootstrapping	none	[48]
Human plasma	none	Correlation feature selection (CFS)	LRA, SVM, RF	K-CV, ROC	Pathway-based metabolite sets analysis (pathifier)	[65]
	Scaling (median value scaled), Transformation (log transformed)	ANOVA, PCA	none	none	none	[66]
	none	T-test, PCA, HCA	PLS-DA, RF	K-CV, ROC	Pathway enrichment analysis (metaboanalyst)	[33]
Human BC cell lines, plasma	none	KS-test, T-test, PCA	none	none	none	[50]
Human saliva	none	none	none	none	none	[52]
	Scaling (Pareto and total intensity value scaled)	T-test, PCA	PLS-DA	ROC, Permutation test	none	[53]
	none	none	LDA	K-CV, ROC	none	[54]
	Scaling (autoscaled and median value scaled), Transformation (cubic root transformed)	MW-test, HCA	PLS-DA, OPLS-DA	MCCV, Permutation test	none	[51]
	Experimental correction (internal standard corrected)	MW-test, PCA	PLS-DA, SVM, LRA	K-CV, ROC	none	[102]
Human tissues	Scaling (Pareto scaled)	PCA	OPLS	K-CV	none	[55]
	none	PCA, HCA	none	none	none	[56]
	Scaling (median scaled), Transformation (log transformed)	T-test	none	none	none	[57]
	Scaling (total intensity value scaled)	T-test	PLS-DA	LOOCV, ROC	Pathway enrichment analysis (metaboanalyst)	[74]
	Scaling (median scaled)	PCA	PLS-DA	LOOCV	none	[75]
	Scaling (median scaled)	T-test, PCA	PLS-DA	LOOCV	none	[78]
	Transformation (log transformed)	T-test, Pearson correlation, HCA	none	none	none	[76]
	none	T-test, Pearson correlation	PLS-DA	K-CV, ROC	none	[77]
Human serum	Centering (mean centered), Scaling (total intensity value scaled)	PCA	PLS-DA, OPLS-DA	K-CV, ROC	none	[67]
	Centering (mean centered), Scaling (total intensity value scaled)	T-test, PCA, ANOVA	OPLS	K-CV, ROC, Bootstrapping	none	[68]
	Transformation (log transformed), Experimental correction (internal standard corrected)	ANOVA, PCA	PLS-DA, LRA	K-CV, ROC	none	[69]
Human urine	Scaling (autoscaled and median value scaled), Transformation (cubic root transformed)	T-test, HCA	PLS-DA, SVM, RF	MCCV, ROC	Pathway enrichment analysis (metaboanalyst)	[59]
Drug therapy	-	-	-	-	-	-
BC cell line	none	T-test	none	none	none	[62]
Human blood	Transformation (log transformed)	T-test, Pearson correlation	none	none	none	[70]
BC tissues	Centering (mean centered), Transformation (log transformed - only in univariate analysis)	T-test, Pearson correlation, PCA	PLS-DA	K-CV, Permutation test	none	[79]
	Scaling (mean scaled - only in PCA)	ANOVA, Spearman correlation, PCA	RF	K-CV, Bootstrapping, Permutation test	none	[80]
	Scaling (total intensity value scaled)	MW-test	OPLS-DA	LOOCV	none	[81]
	none	Spearman correlation	none	none	none	[82]
Serum	Scaling (total intensity value scaled)	T-test	PLS, PLS-DA	LOOCV, ROC	none	[71]
Serum, tissues, cell lines	none	T-test, ANOVA, PCA	PLS-DA	K-CV, ROC	Pathway enrichment analysis (metaboanalyst)	[63]
Urine	Scaling (total intensity value scaled)	KS-test, L-test, SW-test, T-test, PCA	OPLS-DA	K-CV, ROC	Pathway enrichment analysis (metaboanalyst)	[73]
	none	T-test, PCA	PLS-DA	K-CV	none	[84]
Metabolic reprogramming	-	-	-	-	-	-
Human BC cell lines, BC xenografts	none	ANOVA, PCA	PLS-DA	K-CV	none	[64]
Mouse BC tissue	Scaling (median scaled)	ANOVA, PCA	none	none	none	[83]
Endogenous factors	-	-	-	-	-	-
Human plasma	none	T-test, Spearman correlation, PCA	LRA	ROC	none	[31]
Human serum	Transformation (log transformed)	Pearson correlation, PCA	LRA	none	none	[72]

ANOVA – Analysis of variance; ROC – Receiver operating characteristic; LOOCV – leave-one-out-cross validation; AUC – Area under the curve; BFS– Bootstrap feature selection; GGM– Gaussian graphical modelling; HCA – Hierarchical cluster analysis; LDA – Linear discriminant analysis; MCCV – Monte Carlo cross validation; MWT – Mann Whitney U test; NRI – Net reclassification improvement; OPLS-DA – Orthogonal projections to latent structures discriminant analysis; LRA - logistic regression analysis; OSC-PLS – Orthogonal signal correction partial least squares; PC – Pearson correlation; PCA – Principal component analysis; PEA – Pathway enrichment analysis; PLS-DA – Partial least squares discriminant analysis; RF – Random Florest classifier; SCC – Spearman correlation coefficient; SVM – Support vector machine; VIP – variable importance in projection.

## Supplementary Materials

The following is available online at https://www.mdpi.com/2218-1989/9/5/102/s1, Figure S1: (a) Total identified metabolites by analytical technique and (b) number of samples used by each type of biological specimen.
