# Supplementary figures and images for: Breast Cancer Metabolomics: From Analytical Platforms to Multivariate Data Analysis. A Review
Source: Metabolites. 2019 May 22;9(5):102. doi: 10.3390/metabo9050102 (PMC6572290; doi:10.3390/metabo9050102)

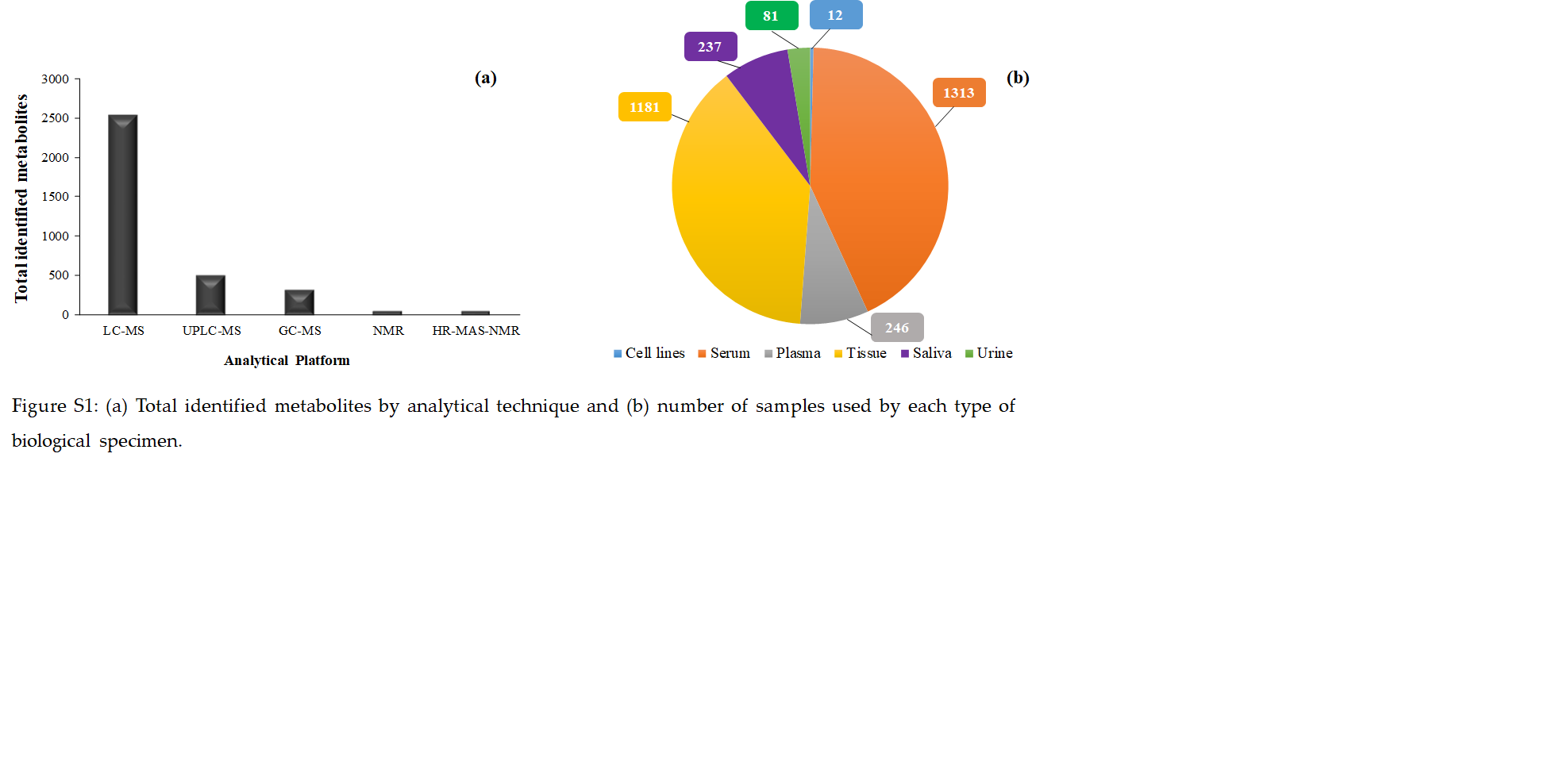

Supplement: Supplementary file 1 [file metabolites-09-00102-s001.zip › metabolites-451707-supplementary.tif]
